# First impressions of a financial AI assistant: differences between high trust and low trust users

**DOI:** 10.3389/frai.2023.1241290

**Published:** 2023-10-03

**Authors:** Simon Schreibelmayr, Laura Moradbakhti, Martina Mara

**Affiliations:** Robopsychology Lab, Linz Institute of Technology, Johannes Kepler University Linz, Linz, Austria

**Keywords:** human-AI interaction, banking, user perception, trust calibration, acceptance, agency, survey

## Abstract

Calibrating appropriate trust of non-expert users in artificial intelligence (AI) systems is a challenging yet crucial task. To align subjective levels of trust with the objective trustworthiness of a system, users need information about its strengths and weaknesses. The specific explanations that help individuals avoid over- or under-trust may vary depending on their initial perceptions of the system. In an online study, 127 participants watched a video of a financial AI assistant with varying degrees of decision agency. They generated 358 spontaneous text descriptions of the system and completed standard questionnaires from the Trust in Automation and Technology Acceptance literature (including perceived system competence, understandability, human-likeness, uncanniness, intention of developers, intention to use, and trust). Comparisons between a high trust and a low trust user group revealed significant differences in both open-ended and closed-ended answers. While high trust users characterized the AI assistant as more useful, competent, understandable, and humanlike, low trust users highlighted the system's uncanniness and potential dangers. Manipulating the AI assistant's agency had no influence on trust or intention to use. These findings are relevant for effective communication about AI and trust calibration of users who differ in their initial levels of trust.

## 1. Introduction

When someone else makes financial decisions for you or suggests how to manage your bank account, it is a highly trust-relevant situation. Financial decisions involve risks and may have long-term implications for the account holder. Therefore, it is important to evaluate how trustworthy those who make or propose such decisions are. If one places too much trust in a financial advisor who actually does not deserve the level of trust, for example due to bad intentions or a lack of competence, this can lead to over-reliance, wrong decisions and associated harms for the trustor (Miller et al., 2016; Robinette et al., 2016; Chong et al., 2022). If, on the other hand, one distrusts a financial advisor despite their good intentions and high level of competence, one may miss out on possible benefits or have to allocate alternative resources to reach comparable results (Grounds and Ensing, 2000; Kamaraj and Lee, 2022).

Today, trust-relevant decisions are increasingly made in conjunction with Artificial Intelligence (AI). AI advisors and other algorithmic decision support systems are used in the field of banking and investments (Day et al., 2018; Shanmuganathan, 2020), but also in many other high-risk domains such as medical diagnoses (Dilsizian and Siegel, 2014; Das et al., 2018), human resources (Vrontis et al., 2022; Chowdhury et al., 2023), mushroom identification (Leichtmann et al., 2023a,b) and even court decisions (Hayashi and Wakabayashi, 2017; Rosili et al., 2021). How to avoid *over-trust*, i.e., unjustified excessive trust in AI systems, and *under-trust*, i.e., insufficient trust in reasonably fair and accurate AI systems, has therefore become a major topic of social scientific, psychological, and ethical research of human-AI collaboration in recent years (Miller et al., 2016; Eigenstetter, 2020). A responsible perspective on human-AI relations can never aim at simply boosting user trust, but rather supporting the calibration of appropriate levels of trust in AI. In this context, *calibrated trust* has been described as trust that is proportionate to the actual trustworthiness (objective capabilities and limitations) of an AI system, rather than trusting excessively or insufficiently (Lee and See, 2004; Boyce et al., 2015; de Visser et al., 2020). Trust calibration involves accurate assessments of an AI system's strengths and weaknesses and adjusting one's subjective level of trust accordingly. Exploring methods for dampening (reducing over-trust) or repairing (reducing under-trust) mis-calibrated trust in AI are therefore of great interest to the scientific community (Lee and See, 2004; de Visser et al., 2020; Chong et al., 2022).

Previous research has found that providing information can contribute to more adequate calibration of trust in AI. By offering more detailed explanations about the strengths and limitations of an AI system, and thus clarifying potential misconceptions and false expectations, users can adjust how much they want to trust the system's output (Boyce et al., 2015; Körber et al., 2018; Miller, 2019; Tomsett et al., 2020; Leichtmann et al., 2023b). At the same time, empirical studies from the field of human-robot interaction indicate that users often have very different initial perceptions and mental models of the same machine and therefore trust or distrust them for very different reasons (Olson et al., 2009; Chien et al., 2016; Matthews et al., 2020). Over the years, hundreds of different cognitive architectures have been described and some of them have been integrated into AI-based models to investigate different approaches, encompassing aspects such as thinking similar to humans, rational thinking, human-like behavior, and rational behavior (Kotseruba et al., 2016; Lieto et al., 2018). While for some users utilitarian dimensions such as the competence, performance, or practicality of the AI system for a specific task might lead to high trust, other users might base their distrust more on social-emotional components such as a low human-likeness or a perceived lack of adherence to social norms. It is likely that the information that could help these users to establish adequate trust would be different.

Consequently, to estimate what kind of explanations about an AI application can effectively contribute to appropriate trust calibration among different user groups, it is important to look at variations in first impressions of the system. The present work therefore investigates how initial perceptions of a financial AI assistant differ between people who report they (rather) trust and people who report they (rather) do not trust the system. In order to capture potentially different user perceptions in a broad and less biased way, we combine typical closed self-report scales with an analysis of qualitative textual descriptions generated by the study participants themselves. We decided to conduct the study in the context of AI in banking, as this is a sensitive domain in terms of risk and vulnerability, making trust a particularly relevant concept (Hannibal, 2023).

### 1.1. AI as financial advisor

AI assistants have established a place in our lives and are applied millions of times worldwide (Statista, 2021), for which a specific and increasing number is taking place in the financial sector and the banking industry (Kochhar et al., 2019). Artificial Intelligence as an advisor in financial decisions is becoming a reality in everyday business (Ludden et al., 2015; Vincent et al., 2015; Jung et al., 2018; Shanmuganathan, 2020). AI can advise on whether to make bank transfers, deposit money, buy stocks, or to make other investments, sometimes in a highly speculative manner (Fein, 2015; Tertilt and Scholz, 2018). For instance, to promptly respond to price fluctuations, many individuals execute such financial decisions directly on their smartphones or tablets (Varshney, 2016; Azhikodan et al., 2019; Lele et al., 2020). Privacy considerations are crucial (Manikonda et al., 2018; Burbach et al., 2019), and the level of autonomy of a financial bot may impact its acceptance and adoption. As financial AI assistants have access to sensitive data and may be able to make critical decisions independently, users may be hesitant to use it if they perceive a lack of control over the system's actions.

### 1.2. Trust in AI

Research on human-machine interaction shows that individuals engage in social behavior toward machines by applying heuristics from interpersonal relations (Nass et al., 1997; Christoforakos et al., 2021). Trust is a critical component in social interactions, as it allows individuals to rely on and believe in the actions and statements of others (Rotter, 1980; Scheuerer-Englisch and Zimmermann, 1997; Neser, 2016). Social psychologists view trust as a multidimensional construct that is composed of cognitive, affective, and behavioral dimensions (Johnson and Grayson, 2005; Righetti and Finkenauer, 2011; Lyon et al., 2015). This means that trust involves positive expectations toward a trustee based on an emotional bond, and it expresses itself in concrete actions of both the trustor and the trustee. Trust also depends on the existence of risk and the individual's willingness to accept vulnerability, and it is associated with expectations of a positive outcome and positive prospective behavior from the other person (Mayer et al., 1995; Flores and Solomon, 1998).

Cognitive trust (as one route of trust formation) is characterized by the assessment of an individual's reliability and dependability (Rempel et al., 1985; McAllister, 1995; Colwell and Hogarth-Scott, 2004), based on the trustor's rational evaluation of the trustee's knowledge, competence, or understandability (McAllister, 1995; Kohn et al., 2021). In contrast, affective trust is an advancement of cognitive trust (Chen et al., 1998; Kim, 2005), which involves mutual interpersonal care and concern, and is characterized by an emotional bond and feelings of security between individuals (Rempel et al., 1985; Johnson and Grayson, 2005). Emotional dependence is a critical determinant in which the impact on trust is more closely associated with personal experiences as compared to cognitive trust. Both the emotional aspect and task-specific abilities may lead to either a positive or negative evaluation of an agent's trustworthiness. Components such as competence and knowledge gained from interpersonal interaction research (McAllister, 1995) are highly important in HCI and affect the level of trust (Chen and Terrence, 2009; Christoforakos et al., 2021) and ultimately the acceptance of automated systems (Ward et al., 2017).

When interacting with digital assistants and AI applications, it may be difficult to accurately evaluate trust-relevant characteristics (Rai, 2020). Often labeled as “black box” services, users have less knowledge about what actually is transpired in the service performance which then requires a high portion of trust (Rosenberg, 1983; MacKenzie, 2005; Devlin et al., 2015). Appropriate trust calibration is one important factor which should be considered in the use of AI powered assistant tools. Low levels of trust can lead to disuse (Lee and Moray, 1992; Parasuraman and Riley, 1997), while very high levels of trust in automated systems may be associated with over-reliance and over-trust (Freedy et al., 2007; Parasuraman et al., 2008; Körber et al., 2018). Additionally, users often evaluate their own decisions in comparison to automated solutions but may also develop a tendency to over-rely on automated systems (Young and Stanton, 2007; Chen and Terrence, 2009). Conversely, people who overestimate their performance tend to exhibit under-reliance on AI systems (similar to the Dunning-Kruger Effect in social psychology), which hinders effective interaction with AI-powered assistant tools (He et al., 2023).

### 1.3. AI agency

In addition to certain voice characteristics such as gender, naturalness or accent (Nass and Brave, 2005; McGinn and Torre, 2019; Moradbakhti et al., 2022; Schreibelmayr and Mara, 2022), other features on the behavioral level of assistance tools like agency are becoming more and more important (Kang and Lou, 2022). Agency pertains to an AI assistant's self-control ability (e.g., Gray et al., 2007), whereas autonomous AI usually has high agency, making decisions based on data, while low agency AI relies on consistent human input. Machines and systems work on a highly autonomous level and according to the situation, offering their help adaptively and in real time (Qiu et al., 2013; Profactor, 2021). Even though users want to experience a proactive style when interacting with a chatbot (Medhi Thies et al., 2017; Pizzi et al., 2021), they also crave some sense of control over the chatbot's or autonomous system's actions and may feel threatened if they behave too autonomously (Złotowski et al., 2017; Stein et al., 2019; Seeber et al., 2020a,b). By way of example, proactivity for irrelevant information is viewed negatively (Chaves and Gerosa, 2021), but it is largely unclear which degree of agency is actually desirable and demanded for AI. Various studies indicate the effects of agency on interactions between humans and virtual characters or robots (Guadagno et al., 2007; Beer et al., 2014; Fox et al., 2015; Pitardi et al., 2021) and Stein et al. (2019) have shown, for example, that participants experienced significantly stronger eeriness if they perceived an empathic character to be an autonomous AI. Considering the phenomenon of the uncanny valley (MacDorman and Ishiguro, 2006; Mori et al., 2012), the concept of a thinking robot that autonomously generates ideas, desires, and expresses needs (Parviainen and Coeckelbergh, 2021; Hanson Robotics, 2023) is unsettling and evokes strong feelings of eeriness alongside fascination (Gray and Wegner, 2012; Stein and Ohler, 2017; Appel et al., 2020). The degree of agency differs depending on technical possibilities and improvements and more research has to be done taking various perspectives into account to create a user-centric point of view. Based on the literature, the degree of agency of an AI banking assistant should be considered in the present study to examine the impact on trust and intention to use.

### 1.4. The present study

In the current research, we asked participants about their perceptions of an artificially intelligent banking assistant. As the utilization of assistance systems in the financial sector is becoming more important and poses potential risks related to trust theories (Mayer et al., 1995; Lee and See, 2004), we chose this particular context to examine participants' impressions after they viewed a video introduction of a fictitious AI banking assistant. We explored impressions that were evoked by the AI banking assistant across different user groups (low vs. high trust) by asking the participants to write down how they would describe the presented AI. Additionally, participants answered closed self-report questionnaires typically used in empirical studies on trust in automation, technology acceptance, and the uncanny valley phenomenon. As experimental manipulation, we incorporated two levels of autonomy into the AI banking assistant. Factors of the technology-acceptance model (TAM, Venkatesh and Bala, 2008) were included and discussed in the light of the Trust in Automation literature (Körber, 2019).

## 2. Methods

The study described in the following was conducted as part of a larger project with multiple research questions. To ensure transparency, we would like to mention that the present study builds upon a preliminary investigation in which the manipulation check failed. Therefore, we strengthened the manipulation by placing additional emphasis on the textual content in the videos (stimulus) and dividing the videos into two parts to maintain the impression created immediately after the manipulation. Below, we give the characteristics of our sample, the study procedure, and the measures (e.g., trust, intention to use) used. For the sake of completeness, other variables that were also surveyed but are not relevant to the present paper are briefly listed: Voice-realism, pleasantness, perception of risk, tolerance of ambiguity, and desirability for control.

### 2.1. Sample size justification and participants

The sample size required for the present online experiment was calculated by a power analysis, which helps to determine how many study participants are needed, using G^*^Power (Cohen, 1992; Faul et al., 2007). For the calculation, a medium effect size of *d* = 0.50 was assumed and α error probability was set to 0.05. In order to achieve a power (1 – β) of 80% (β represents the probability of committing a Type II error, which is the error of failing to reject a null hypothesis that is actually false), the analysis resulted in a recommended sample size of at least *N* = 128 to run a test procedure with two independent groups. The participants were recruited through a snowball approach^1^ at the campus of the Johannes Kepler University Linz, Austria. Overall, 136 participants took part in the online experiment. After excluding 9 participants as their indicated age was below 16 years or because of insufficient German skills, the final sample consisted of 127 participants (62 females, 61 male, 2 non-binary, 2 no specification), aged between 17 and 82 years (*M* = 30.55, SD = 15.47). 49.6% of participants had completed compulsory education with a high school diploma (Matura or Abitur), 38.6% had completed university education with a bachelor's level or higher, and 11.8% had some other type of vocational training.

### 2.2. Study design and procedure

The experiment was conducted as a 2 x 1 between-subjects design, involving 2 groups of participants experiencing distinct levels of the independent variable to analyze its effects on the dependent variable (*N*_Highagency_ = 61, *N*_Lowagency_ = 66), and was designed with the online survey software (Questback, 2020). At the beginning of the study, all participants were instructed to either use headphones or keep their computer/laptop audio on a high volume for the duration of the study. After that, participants read an introduction, confirmed their consent, filled out demographic information (including age, gender, and level of education) and were asked to fill in personality questionnaires (openness to experience, neuroticism, and propensity to trust). Once these scales were completed by the participants, brief instructions appeared followed by one out of two AI banking assistant videos, which were randomly assigned to each participant. Additionally, every video was divided into two parts (~1 min each). Subsequent to the first part, the participants were queried about the degree of human-likeness and eeriness of the AI banking assistant. Items regarding AI banking assistant's agency (manipulation-check) were asked after the first part of the video as well. After the second part of the video, the participants were requested to assess the perceived level of trustworthiness they had in the assistant featured in the video. Then, the participants were requested to write down attributes of the AI banking assistant and their intention to use it. Finally, some check items were asked to make sure that the participants had clearly understood the sound of the video (it was technically not possible to skip the video), that their German language skills allowed them to understand the content of the study, and that they had answered all questions honestly and conscientiously. The average processing time for the entire survey was approximately 15 min and 31 s.

### 2.3. Stimulus material and manipulations

#### 2.3.1. AI banking assistant videos

**Figure 1:**
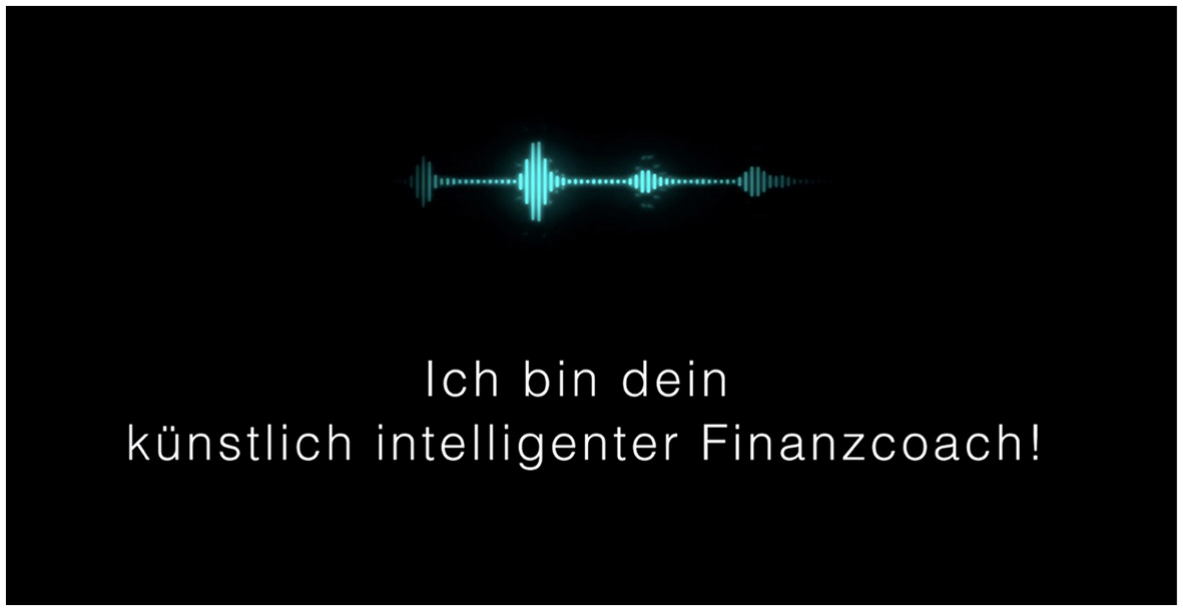
Screenshot from an AI banking assistant video including a text passage (English translation: “I am your artificially intelligent financial assistant”) and sound waves.

We used two videos, differing in the spoken content (low/high agency). The videos were created using the software Adobe After Effects. In order to make the respective condition (low/high agency) stand out, certain content, words and phrases of the spoken text were visualized and highlighted in the video (e.g., “at your request” = low agency, vs. “without your intervention” = high agency). The voice for the AI banking assistant was created with the help of the text-to-speech online platform ttsmp3.com, powered by AWS Polly (Amazon Web Services), based on the default settings of the voice “Vicki”. A sound wave was shown, to visualize the AI banking assistant's voice in the video (see Figure 1). The total length of the video was 1 min and 53 s in the high agency condition and 1 min and 52 s in the low agency condition (each video was divided into two parts as we asked some scales in between).

#### 2.3.2. AI banking assistant agency (manipulations)

In the context of our study, “high agency” refers to AI systems that can plan ahead and make decisions without user command, while “low agency” AI relies on consistent human input for tasks and choices. High agency AI operates independently, low agency AI requires ongoing human guidance. We varied the AI banking assistant's agency level between high and low agency through manipulating its textual introduction. In the low agency condition, for example, the AI banking assistant offered: “If you connect me with your account information, I can support you with deposits, planned savings and other tasks.” In the high agency condition, the same service was introduced in the following manner: “I will autonomously connect myself with your account information. Without effort on your part, I will take care of deposits, planned savings and other tasks.” Overall, 6 phrases were adapted and differed between the conditions to achieve the agency level manipulation.

### 2.4. Measures

*Open-ended descriptions of the AI banking assistant*. We asked the participants to provide a verbal description of the AI banking assistant using three terms in an open comment format. (“If you were to tell someone else about the AI banking assistant in the video, what terms would you use to describe it?”).

*Trust* was measured with two items of the trust in automation subscale (Körber, 2019). The items were modified slightly to match the content of the AI banking assistant (e.g., “I would trust the AI banking assistant.”, ranging from 1 = *not at all* to 5 = *very much*) (Cronbach's α = 0.760). Find all items in Appendix B.

*Competence* (4 items, Cronbach's α = 0.598), *understandability* (2 items, Cronbach's α = 0.744), and *intention of developers* (2 items, Cronbach's α = 0.738) were measured with the help of shortened scales taken from the trust in automation model (Körber, 2019). The items were modified slightly to match the content of the AI banking assistant. Find all items in Appendix B.

*Intention to use* was measured with the help of two items based on the intention to use items from the Technology Acceptance Model (TAM3, Venkatesh and Bala, 2008). A 5-point Likert-scale (1 = *not at all* to 5 = *very much*) was used and the items were slightly adapted to fit the context of the current experiment: “I could imagine using the AI banking assistant in the future.” and “I would like to be informed about products that are similar to the AI banking assistant.”. The reliability of the items was high, with a Cronbach's α of 0.804.

*Human-likeness* of the AI banking assistant, which refers to the extent to which a system resembles human attributes, behavior, appearance, or cognitive capabilities, was assessed with five items on a five-point semantic differential scale [e.g., 1 = *synthetic*, 5 = *real*; 1 = *mechanical*, 5 = *organic*, adapted from Ho and MacDorman (2010), which yielded an excellent reliability with Cronbach's α = 0.832.]

*Uncanniness* of the AI banking assistant, which describes an unsettling sensation or a sense of discomfort and creepiness (e.g., Mori, 1970; Mori et al., 2012; due to the blurring of human and non-human traits referring to the uncanny hypothesis), was measured with three items on a five-point semantic differential scale [e.g., 1 = *scary*, 5 = *comforting*, as example of an inverse coded item, adapted from Ho and MacDorman (2010), Cronbach's α = 0.837].

*Agency*, consisting of two items, was used as a manipulation check for the different agency levels (low/high) of the two conditions (e.g., “The AI banking assistant has the ability to act in a self-controlled manner”; Cronbach's α = 0.725). A 5-point Likert-scale was used.

We used the two dimensions of the Big Five personality traits, *openness to experience*, which refers to an individual's inclination to engage with novel ideas, experiences, and intellectual or artistic pursuits (Neyer and Asendorpf, 2018) and *neuroticism*, which is characterized by a tendency toward experiencing negative emotions such as anxiety, depression, moodiness, and emotional instability (Neyer and Asendorpf, 2018). Both scales were explained by three items each, based on the 15-items short-scale (5 dimensions) from the Socio-Economic Panel (SOEP, see Schupp and Gerlitz, 2014). A 5-point Likert-scale was used. Internal consistencies were good (openness to experience: Cronbach's α = 0.708, neuroticism: Cronbach's α = 0.713).

*Propensity to trust*, as a personality trait characterized by an individual's innate inclination to believe in the reliability and good intentions of others in various situations (Patent and Searle, 2019), was assessed with the help of three items (5-point Likert-scale) taken from the trust in automation model (Körber, 2019), showing acceptable internal consistency (Cronbach's α = 0.503).

## 3. Results

Before analysis, we examined if the prerequisites of parametric analyses (normal distribution, homoscedasticity of the variances) were met by our data. As this was not the case for several variables, we decided to apply non-parametric test procedures. Therefore, we used the Wilcoxon-Mann-Whitney-Test to compare the two independent groups and determined if their distributions significantly differs, to assess whether one group tends to have higher or lower values than the other based on the ranking of observations in the samples. Zero-order correlations (Spearman's rank) between all variables were computed and can be found in Appendix Table 1.

**Figure 2:**
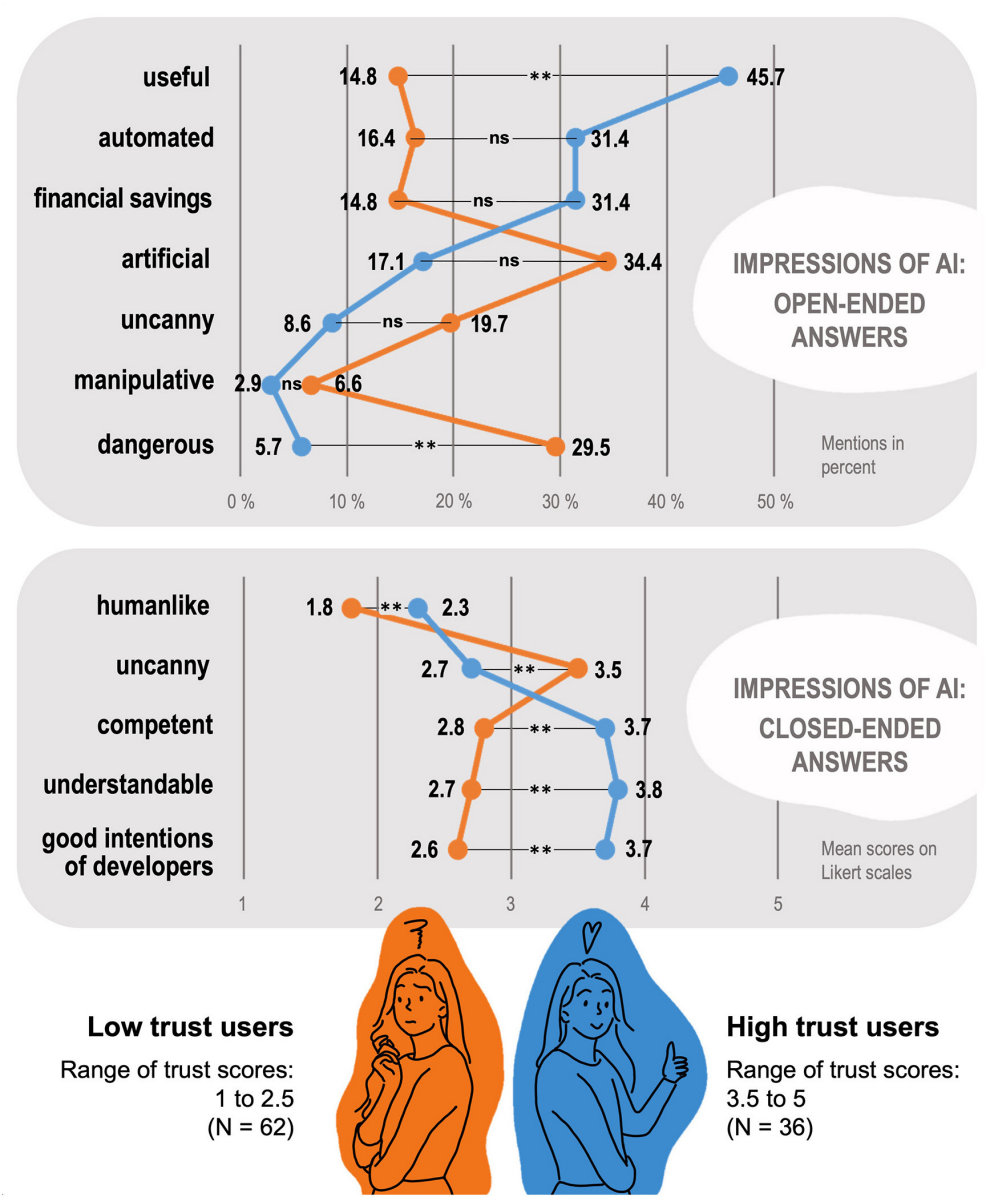
Overview of characteristics attributed to the financial AI assistant by users with low trust and users with high trust in the system. Open-ended descriptions represent spontaneous characterizations that users generated themselves, indicated in percentage frequency per group. Closed-ended descriptions represent answers to preset questionnaires, indicated in mean scores per group. Statistical significance of group differences: **p < 0.001; ns = non-significant.

Our analysis aimed to compare individuals who exhibit low levels of trust in the AI banking assistant with those who have high levels of trust in it. To accomplish this, we removed participants who provided an average value of 3 on the 5-point Likert-scale for trust in the further evaluation and formed the two extreme groups. All participants with trust scores ranging from 1 to 2.5 were placed in the low trust group, while those with trust scores ranging from 3.5 to 5 were placed in the high trust group (1.00 = 10 VPN, 1.50 = 8 VPN, 2.00 = 26 VPN, 2.50 = 18 VPN, 29 VPN with a score of 3.00 were excluded, 3.50 = 20 VPN, 4.00 = 11 VPN, 4.50 = 3 VPN, 5.00 = 2 VPN). Figure 2 provides a descriptive representation of the two groups, low trust and high trust. The corresponding statistical values (e.g., standard errors of means) can be found in Appendix Table 2.

In further analysis, we compared open-ended descriptions of the AI banking assistant (user-generated impressions) and closed-ended scale means (e.g., intention to use) between these two extreme groups. Additionally, we investigated whether there were any differences in the measured personality dimensions between the two user groups. Finally, we tested whether there were any differences in the variables between the two different agency levels (low vs. high) of the AI banking assistants as well. Since the degree of agency (high/low) was manipulated, we also examined the distribution of the two conditions within the two groups low trust vs. high trust. The descriptive analysis indicates that the two agency variants are almost equally distributed within the two groups low trust and high trust, *X*^2^(1,98) = 0.70, *p* = 0.791.

### 3.1. Analysis of open-ended descriptions

After the participants wrote down three terms (e.g., nouns, adjectives) that described the AI banking assistant in an open comment field, two to three independent raters evaluated these terms. In total, 358 (23 missing) descriptions were recorded. The raters had to determine for each participant whether at least one of the three terms reflected specific categories (useful, automated, financial savings, artificial, uncanny, manipulative, dangerous).

These seven categories were created collaboratively *post-hoc* based on the most frequently occurring categories identified. Per category, each user-generated answer was assigned either 0 (if none of the terms matched the category) or 1 (if at least one of the terms matched the category). In cases where the raters did not agree, a decision was made collaboratively. The interrater reliabilities demonstrated substantial to nearly perfect agreement (Landis and Koch, 1977): Useful: κ = 0.727, automated: κ = 0.977, financial savings: κ = 0.832, artificial: κ = 0.895, uncanny: κ = 0.899, manipulative: κ = 0.725, dangerous: κ = 0.634.

Furthermore, the raters assigned a separate category for an overall impression (sentiment) to each test participant. They rated this category based on whether the terms used were only neutral, at least one positive, at least one negative, or a combination of both negative and positive terms. The sentiment was rated on a scale from −1 (negative) to 0 (neutral) to 1 (positive), depending on the terms used (14 ambivalent combinations including both negative and positive terms were excluded from the analysis, 5 VPN were additionally excluded due the fact that they didn't write down any descriptions). Subsequently, a mean score was calculated based on these evaluations, which represented the overall impression (sentiment) and was utilized for comparing the different user groups (low vs. high trust) in further analysis.

We conducted a chi-square test to determine whether there was a significant difference between the two groups (low trust vs. high trust) across the seven aforementioned categories (useful, automated, financial savings, artificial, uncanny, manipulative, dangerous). The percentage frequency of term occurrences for the categories useful, *X*^2^(1,96) = 11.068, *p* < 0.001, ϕ = 0.34, and dangerous, *X*^2^(1,96) = 7.634, *p* = 0.006, ϕ = −0.28, show significant differences between the low trust and high trust group. People who trusted the AI banking assistant perceived fewer risks and danger (5.7%) in using it, as opposed to the user group that distrusts the assistant, and thus used more terms to describe the potential dangers of its use (29.5%; e.g., “deceptive”, “suspect”). Similarly, the group that trusted the AI banking assistant regarded it as useful (45.7%; e.g., “optimization”, “workload reduction”) whereas a substantial number of those who distrusted the AI banking assistant viewed its assistance as less useful (14.8%) and, as previously stated, risky and dangerous (29.5%).

In the remaining five categories, no statistically significant differences can be found, whereby three of them reach a borderline area on an alpha level of 0.05: Automated, *X*^2^(1,96) = 2.942, *p* = 0.086, ϕ = 0.175; financial savings, *X*^2^(1,96) = 3.749, *p* = 0.053, ϕ = 0.198; artificial, *X*^2^(1,96) = 3.286, *p* = 0.070, ϕ = −0.185; uncanny, *X*^2^(1,96) = 2.079, *p* = 0.149, ϕ = −0.147; manipulative, *X*^2^(1,96) = 0.617, *p* = 0.432, ϕ = −0.080. Approximately 20% of the participants belonging to the low trust group perceived the AI banking assistant as uncanny, and around 34% associated terms such as “artificial” with the assistant. Conversely, in the high trust group, the number of participants who described the AI banking assistant as scary is just under 9% and only around 17% described the assistant as artificial. Furthermore, in the high trust group, ~31%—more than twice the number in the low trust group (15%)—associated the assistant with financial savings.

Comparing the mean sentiment score (overall impression) between the low trust (*M* = −0.48) and high trust (*M* = 0.39) groups also showed a significant difference. Specifically, participants who trusted the AI banking assistant exhibited a significantly more positive attitude (e.g., “practical”, “expectant”) toward the assistant compared to those who distrusted it. Conversely, the latter group demonstrated a negative attitude, as evidenced by their use of negative associations and terms toward the AI banking assistant (e.g., “unnecessary”, “dubious”).

### 3.2. Analysis of closed-ended scales

In the subsequent analysis, we compared the two user groups low trust vs. high trust based on the scale indices of the variables. Wilcoxon-Mann-Whitney tests revealed significant differences in mean values for the following variables: Trust (*U* = 0.00, *Z* = –8.378, *p* < 0.001, *r* = 0.74), intention to use (*U* = 353.50, *Z* = –5.687, *p* < 0.001, *r* = 0.50), competence (*U* = 323.00, *Z* = –5.880, *p* < 0.001, *r* = 0.52), understandability (*U* = 313.50, *Z* = –6.016, *p* < 0.001, *r* = 0.53), intention of developers (*U* = 318.50, *Z* = –5.959, *p* < 0.001, *r* = 0.53), human-likeness (*U* = 631.00, *Z* = –3.592, *p* < 0.001, *r* = 0.32), and uncanniness (*U* = 615.50, *Z* = –3.713, *p* < 0.001, *r* = 0.33). Except for the variable uncanniness, the participants from the high trust group show higher mean values in all variables. In the variable uncanniness, the mean value from the low trust group (M = 3.52, SD = 0.85) is significantly higher compared to the high trust users (M = 2.71, SD = 0.96). All means and standard deviations including all participants (*N* = 127) can be found in Appendix Table 1. All means calculated separately for the two user groups (high trust vs. low trust) are shown in Figure 2.

### 3.3. Analysis of individual differences

There was no significant difference observed between the low trust and high trust user groups regarding the variables neuroticism (*U* = 965.50, *Z* = –1.118, *p* = 0.264, *r* = 0.10) and openness to experience (*U* = 1,108.50, *Z* = –0.056, *p* = 0.955, *r* = 0.05). The personality trait propensity to trust (*U* = 546.50, *Z* = –4.264, *p* < 0.001, *r* = 0.38), on the other hand, differed significantly between the two groups. Specifically, participants who displayed a high level of trust in the financial assistant also had a higher tendency to trust in general, as compared to those who displayed low levels of trust in the AI banking assistant. No significant differences were found between the low trust vs. high trust group in terms of the distribution of age (*U* = 972.00, *Z* = –1.064, *p* = 0.288, *r* = 0.09) and gender (*U* = 875.00, *Z* = –1.559, *p* = 0.119, *r* = 0.13).

### 3.4. Analysis of group differences with regard to agency (manipulation)

To verify the effectiveness of our manipulation of the two conditions (high/low), participants were asked to rate the level of autonomy exhibited by the AI banking assistant using the agency scale. We computed the mean values and detected significant differences between the groups (*U* = 687.00, *Z* = –6.451, *p* < 0.001, *r* = 0.57), indicating a successful manipulation of the conditions. The high agency bot was perceived as a highly autonomous assistant, while the low agency bot was perceived as a less autonomous assistant.

However, a subsequent analysis investigating differences in levels of trust (*U* = 1,943.00, *Z* = –0.343, *p* = 0.732, *r* = 0.03) and intention to use (*U* = 1,943.50, *Z* = −0.339, *p* = 0.735, *r* = 0.03) showed no significant differences between the low and high agency condition. Furthermore, no significant differences were observed between the low and high agency conditions in terms of the other variables, competence (*U* = 1,822.00, *Z* = –0.928, *p* = 0.353), understandability (*U* = 1,860.00, *Z* = –0.753, *p* = 0.452, *r* = 0.07), intention of developers (*U* = 1,973.00, *Z* = –0.196, *p* = 0.845, *r* = 0.02), human-likeness (*U* = 1,922.50, *Z* = –0.439, *p* = 0.661, *r* = 0.04), and uncanniness (*U* = 1,722.00, *Z* = –1.414, *p* = 0.157, *r* = 0.13).

## 4. Discussion

The findings indicate significant differences between the two groups of users, low trust vs. high trust, which we discovered through open-ended and closed-ended questionnaires. The analysis revealed that the perceptions and attitudes toward the AI banking assistant varied significantly based on the user groups, regarding its potential benefits (useful) and the perceived possibility of harm (dangerous). Specifically, the high trust group acknowledged the financial assistant's usefulness and viewed it as a valuable tool that could assist with tasks, while being less worried about the potential risks associated with it. In contrast, individuals who were skeptical of the AI banking assistant perceived only little assistance from the bot and instead saw the significant dangers that it could pose. In examining the other categories, such as manipulation and uncanniness, there were no significant differences in the frequency of terms between the two user groups. However, the participants of the low trust group perceived the AI banking assistant as particularly scary (almost 20%). Additionally, 16% of the users of the low trust group perceived the AI banking assistant as automated and approximately 34% as artificial.

Consistent with the literature on trust in automation (Körber, 2019), competence was found to be essential and has a significant impact on the level of trust among all participants. Additionally, usefulness, as one of the two key components in the Technology Acceptance Model (Venkatesh and Bala, 2008), proves to be a determining factor in influencing the intention to use the AI banking assistant, as evidenced by the difference between the two user groups. The strong correlations between competence and trust are apparent in the z-order correlations. To increase trust, one could highlight the system's competence to the low trust group. Additionally, a developer's benevolent intentions can positively impact the level of trust and play a crucial role in the trust calibration process.

The analysis of the closed-ended scales revealed significant differences between the two user groups in all dependent variables. However, unlike the open-ended comments, those who had a lack of trust in the AI system, perceived it as more uncanny and less human-like. Low trust users perceived the AI banking assistant as less competent and the developers' intentions as less benevolent. They also had a lower understanding of the system's workings and calculations, resulting in correspondingly lower intentions to use the tool. Conversely, in the high trust group, all variables, except uncanniness, showed significantly higher values. The AI banking assistant was perceived as highly competent, and users believed that the developers had benevolent intentions. Additionally, users largely understood how the assistant made decisions and took appropriate actions, which increased trust and contributed to a high intention to use the tool.

To establish an effective trust calibration, it is important to address the concerns of both user groups and adjust the level of trust accordingly (Lee and Moray, 1992; Boyce et al., 2015). For the low trust group, it would be beneficial to provide detailed information about the assistant's functions, workings, and calculations to enhance understandability, which directly correlates with trust. By providing information on how the system makes recommendations and decisions, the “black-box” phenomenon could be avoided (e.g., Guidotti et al., 2019). Conversely, the high trust group may be at risk of over-trusting and underestimating the potential dangers of AI powered tools, including autonomous driving or other high-risk applications. Here, clarifying possible risks could facilitate necessary trust calibration to prevent the use of overly risky tools.

In this context, explainable AI (XAI) plays a major role in developing AI systems that provide understandable explanations for their output. Considering these objectives, AI systems should become more transparent and interpretable to users, which is particularly crucial in critical applications where trust, accountability, and adherence to regulations are indispensable (Ehsan et al., 2021). XAI methodologies are typically applied to enhance the interpretability of black-box models for lay human users and to allow them to better assess the trustworthiness of a system or its output (Guidotti et al., 2019; Alicioglu and Sun, 2022; Leichtmann et al., 2023a,b). Although there are models that possess inherent explainability (i.e., models characterized by a significant level of transparency; e.g., Barredo Arrieta et al., 2020), they frequently exhibit the drawback of yielding less precise outcomes. Consequently, *post-hoc* explanation techniques are harnessed to expound upon extant models (e.g., Dosilovic et al., 2018). According to this, requirements collectively aim to strike, among other things, a balance between transparency (AI models' decision-making processes and internal mechanisms), accuracy and performance (not compromise the accuracy and performance of the AI models), and user-centricity (explanations generated should be tailored to the cognitive abilities and needs of the target audience), thereby fostering trust and confidence in AI technologies (Dosilovic et al., 2018; Barredo Arrieta et al., 2020). Modern emotional AI, by recognizing and adapting to user emotions (Lausen and Hammerschmidt, 2020; Li and Deng, 2022), enhances AI system responses for personalized interactions, may foster higher levels of trust through empathetic and contextually aware communication. This may also contribute to improved user satisfaction, engagement, and long-term reliability in human-machine interactions (e.g., Vishwakarma et al., 2021), but it could also generate skepticism or even fuel overtrust in AI-powered tools (e.g., Grill and Andalibi, 2022).

It is interesting that there were no differences in the variables based on the level of agency of the AI banking assistant (manipulation). This suggests that whether the assistant acted more or less autonomously did not matter. Contrary to our expectations based on the uncanny valley hypothesis (Gray and Wegner, 2012; Mori et al., 2012; Appel et al., 2020), the degree of autonomy, especially in the field of banking, seems to have no impact on the sense of uncanniness experienced. This could be attributed to the fact that our study did not depict a thinking robot capable of generating own ideas, possessing desires, or expressing needs autonomously. Instead, it presented an AI banking assistant as a tool utilizing mathematical calculations to provide savings recommendations and perform other “factual” tasks, devoid of emotional elements. Whereas, what mattered was whether the bot was generally perceived as useful or potentially hazardous. One might expect that individuals with higher levels of neuroticism would perceive greater risks associated with the bot, leading to higher neuroticism scores in the low trust group. However, this was not observed, and the distribution of openness to experience scores between the two user groups was the same. In contrast, the personality trait propensity to trust shows a significant difference between the two user groups. The propensity to trust score is significantly higher in the high trust group, suggesting that trust calibration efforts should be tailored to individual user groups. Consistent with previous research, it is important to consider implications for transparency when developing different AI powered tools for various personalities and user groups (Chien et al., 2016). The advantageous use of an AI powered system could be emphasized and providing information could be used to mitigate unfounded risks and dangers.

Distinctions between users who trust and those who distrust a particular system can manifest in various ways, including behaviors, attitudes, and perceptions. It seems that individuals who trust tend to have a more positive view on AI powered tools and are more likely to engage with the system. On the other hand, those who distrust may exhibit negative attitudes and behaviors toward the system, including avoidance or even actively working against it (e.g., “just useless”, “attempted fraud”). Another distinction is that individuals who distrust may be more critical of the system, scrutinizing its actions and decisions more closely. They may also be more hesitant to share personal information or participate in activities that involve the system. In contrast, those who trust may be more willing to provide personal information and engage in activities that involve the system, believing that their information will be handled responsibly. Overall, these differences between the two user groups who trust, and distrust can have significant implications for the success and effectiveness of the system in question. Understanding these distinctions and working to address them can help build trust and improve user experiences.

## 5. Limitations and outlook

In terms of trust calibration, it has been found that the two distinct groups of low trust and high trust are appropriate. However, in a larger sample, it would be beneficial to further investigate the individual differences and personality traits among the participants to adopt a more user-centered approach. The present study did not document any pre-experience with an AI banking assistant or AI powered technology in general, which could potentially impact the development of trust. In subsequent surveys, greater emphasis and analysis should be placed on financial literacy. Since the spontaneous reactions (open-ended descriptions) were only asked at the end of the questionnaire, this represents a further limitation and should be considered in subsequent studies. The study was conducted within the context of financial savings, which, in line with classic trust theories, entails potential risks and dangers. Of course, there are numerous other contexts in which similar conditions apply, and it would therefore be worthwhile to examine the different approaches of user groups in other contexts, such as autonomous driving, more closely.

Various AI-powered tools utilize statistical models to analyze extensive data, learning patterns and connections among words and phrases, with the goal of simulating human intelligence and behavior (Bostrom and Yudkowsky, 2014; Hanson Robotics, 2023; Kosinski, 2023). The choice of these tools, like deep learning or genetic algorithms, may also have the potential to shape perceptions and impressions people have, thereby influencing trust (e.g., Wang et al., 2023). Notably, prominent language models like OpenAI's ChatGPT, Meta's LLaMA, or Google's PaLM2 may vary in attributes like fairness, accuracy, or reliability (Chang et al., 2023; Kaddour et al., 2023; Open AI, 2023). The quality of AI inference, referring to a model's capability to generate predictions or responses based on context and input, may also impact users' reliance on AI (e.g., Toreini et al., 2020). Although this aspect wasn't explored in our current study, it presents a potential avenue for future investigations into the alignment of objective and subjective trustworthiness. Establishing trust hinges on AI producing reliable and unbiased outcomes, particularly in collaborative human-AI decision contexts. Designing AI systems for accuracy, fairness, and transparency thus contributes to strengthening user confidence.

## 6. Conclusion

In this study, we explored initial attitudes and perceptions toward an AI-powered financial assistant, differentiating between two distinct user groups: Those who, after only brief exposure to the system, indicate that they trust it and those who state that they do not. Our findings highlight significant variations in group characteristics and user perceptions. While high trust users acknowledged the AI assistant's usefulness for banking tasks and were less concerned about associated risks, low trust users perceived limited assistance value, found the system uncanny and difficult to understand, and emphasized potential dangers. These insights underscore the importance of recognizing diverse user groups and trust levels in the development of AI-powered tools for banking and beyond. By tailoring trust calibration strategies to different first impressions of the same system, a potential underestimation of risks among users with high initial trust can be more effectively addressed, while unwarranted distrust in reliable systems can also be countered.

## Data availability statement

The original contributions presented in the study are included in the article/Supplementary material, further inquiries can be directed to the corresponding author.

## Ethics statement

This research complied with the tenets of the Declaration of Helsinki, the ethical guidelines of the APA Code of Conduct, local legislation, and institutional requirements. Digital informed consent was obtained from each participant.

## Author contributions

SS organized the database, performed the statistical analysis, and wrote the first draft of the manuscript. All authors contributed to conception and design of the study, manuscript revision, read, and approved the submitted version.

## Footnotes

^1^Individuals who had already participated were asked to invite new study participants. Persons who had already participated in the online-experiment were sensitized to not communicate any additional information about the contents of the study to newly recruited persons.
