# Supplementary material for: First impressions of a financial AI assistant: differences between high trust and low trust users
Source: Front Artif Intell. 2023 Oct 3;6:1241290. doi: 10.3389/frai.2023.1241290 (PMC10579608; doi:10.3389/frai.2023.1241290)
Supplement: Supplementary file 1 [file Data_Sheet_1.docx]

**APPENDIX A**

**Table 1**

| *Zero-order correlations (Spearman´s rho)* | | | | | | | | | | | | | | |  |  |  |  |  |  |
| --- | --- | --- | --- | --- | --- | --- | --- | --- | --- | --- | --- | --- | --- | --- | --- | --- | --- | --- | --- | --- |
| **Measures** | ***M*** | ***SD*** | **1.** | **2.** | **3.** | **4.** | **5.** | **6.** | **7.** | **8.** | **9.** | **10.** | **11.** | **12.** | **13.** | **14.** | **15.** | **16.** | **17.** | **18.** |
| **1. Competence** | 3.20 | .68 | .598 |  |  |  |  |  |  |  |  |  |  |  |  |  |  |  |  |  |
| **2. Understandability** | 3.14 | .90 | .565** | .744 |  |  |  |  |  |  |  |  |  |  |  |  |  |  |  |  |
| **3. Intention of**  **developers** | 3.05 | .92 | .423** | .512** | .738 |  |  |  |  |  |  |  |  |  |  |  |  |  |  |  |
| **4. Trust** | 2.70 | .93 | .615** | .624** | .642** | .760 |  |  |  |  |  |  |  |  |  |  |  |  |  |  |
| **5. Intention to use** | 2.48 | 1.09 | .510** | .422** | .479** | .590** | .804 |  |  |  |  |  |  |  |  |  |  |  |  |  |
| **6. Human-likeness** | 1.96 | .79 | .273** | .361** | .342** | .319** | .348** | .832 |  |  |  |  |  |  |  |  |  |  |  |  |
| **7. Uncanniness** | 3.20 | .93 | -.398** | -.409** | -.399** | -.377** | -.339** | -.308** | .837 |  |  |  |  |  |  |  |  |  |  |  |
| **8. Openness to**  **experience** | 4.08 | .73 | -.032 | -.037 | -.078 | -.038 | -.019 | -.105 | .223* | .708 |  |  |  |  |  |  |  |  |  |  |
| **9. Neuroticism** | 3.07 | .82 | -.077 | -.120 | -.111 | -.088 | -.026 | -.102 | .202* | .240** | .713 |  |  |  |  |  |  |  |  |  |
| **10. Propensity to**  **trust** | 3.04 | .59 | .386** | .286** | .287** | .424** | .394** | .267** | -.402** | -.099 | -.138 | .503 |  |  |  |  |  |  |  |  |
| **11. Useful** | .26 | .44 | .336** | .261* | .252** | .311** | .420** | .220* | -.325** | .080 | -.102 | .210* | .735 |  |  |  |  |  |  |  |
| **12. Automated** | .24 | .43 | .212* | .210* | .323** | .208* | .107 | .117 | -.300* | -.101 | .102 | .181* | .017 | .977 |  |  |  |  |  |  |
| **13. Financial savings** | .22 | .42 | .209* | 207* | .327** | .234** | .164 | .154 | -.144 | -.208* | .040 | .169 | .176 | .307** | .832 |  |  |  |  |  |
| **14. Artificial** | .28 | .45 | -.280** | -.237** | -.018 | -.160 | -.137 | -.202* | -.129 | -.087 | -.053 | -.170 | -.204* | -.086 | -.196* | .895 |  |  |  |  |
| **15. Uncanny** | .15 | .36 | -.132 | -.114 | -.206* | -.149 | -.156 | -.028 | .505** | .052 | .185* | -.216* | -.196* | -.131 | -.172 | -.063 | .899 |  |  |  |
| **16. Manipulative** | .04 | .20 | -.090 | -.156 | -.120 | -.119 | -.060 | -.071 | .117 | .205* | .018 | -.112 | -.029 | -.114 | -.010 | .057 | .140 | .725 |  |  |
| **17. Dangerous** | .19 | .39 | -.209* | -.303** | -.327** | -.362** | -.246** | -.187* | .152 | .013 | .033 | -.151 | -.240** | -.217* | -.254* | .077 | .083 | .324** | .634 |  |
| **18. Gender** | 1.50 | .50 | -.048 | -.002 | -.142 | -.121 | .046 | -.048 | -.236** | -.340** | -.229* | -.013 | .031 | .063 | -.037 | .063 | -.234* | -.117 | .063 | - |
| **19. Age** | 30.55 | 15.47 | .131 | .062 | .038 | .144 | .080 | .208* | -.162 | -.108 | -.145 | .063 | .157 | .058 | .153 | -.216* | -.116 | -.112 | -.036 | .169 |
| *. The correlation is significant at α = 0.01 (two-sided). *. The correlation is significant at α = 0.05 (two-sided).  The diagonal shows the reliability coefficients (Cronbachs alpha for scales, Fleiss´ kappa for interrater reliability).  Variables 1 – 5 and variables 8 - 10: Five-point Likert-scale. Variables 6 – 7: Five-point semantic differential scale. Variables 11 – 17: Dummy coded with 0 (if none of the terms matched the category) and 1 (if at least one of the terms matched the category).  Gender (*N*=123) was dummy coded 0 for men and 1 for women (excluding data of two non-binary participants and two participants who did not report their gender).  *N*s of variables 11 - 17 range from 118 (due to missing values) to 127. | | | | | | | | | | | | | | | | | | | | |

**Table 2**

| *Group differences between Low trust users vs. High trust users* | | | | | | | |
| --- | --- | --- | --- | --- | --- | --- | --- |
| **Measures** | **Low trust users** | | |  | **High trust users** | | |
|  | **Mean** | **Std.-error** | **SD** |  | **Mean** | **Std.-error** | **SD** |
| **1. Useful**** | .15 | .05 | .36 |  | .46 | .09 | .51 |
| **2. Automated** | .16 | .05 | .37 |  | .31 | .08 | .47 |
| **3. Financial savings** | .15 | .05 | .36 |  | .31 | .08 | .47 |
| **4. Artificial** | .34 | .06 | .48 |  | .17 | .06 | .38 |
| **5. Uncanny** | .20 | .05 | .40 |  | .09 | .05 | .28 |
| **6. Manipulative** | .07 | .03 | .25 |  | .03 | .03 | .17 |
| **7. Dangerous**** | .30 | .06 | .46 |  | .06 | .04 | .24 |
| **8. Humanlike**** | 1.80 | .11 | .86 |  | 2.26 | .12 | .72 |
| **9. Uncanny**** | 3.51 | .11 | .85 |  | 2.71 | .16 | .96 |
| **10. Competent**** | 2.85 | .08 | .63 |  | 3.69 | .09 | .54 |
| **11. Understandable**** | 2.69 | .11 | .85 |  | 3.82 | .10 | .58 |
| **12. Good intentions of**  **developers**** | 2.59 | .10 | .81 |  | 3.72 | .11 | .68 |
| **13. Trust**** | 1.92 | .07 | .51 |  | 3.82 | .07 | .43 |
| Statistical significance of group differences: **p < .001  Variables 1 – 7: Dummy coded with 0 (if none of the terms matched the category) and 1 (if at least one of the terms matched the category). Variables 8 - 9: Five-point semantic differential scale. Variables 10 – 13: Five-point Likert-scale.  Low trust users (*N* = 62): Range of trust scores: 1 to 2.5.  High trust users (*N* = 36): Range of trust scores: 3.5 to 5. | | | | | | | |

**APPENDIX B**

***Trust scales*** *(based on the TiA model proposed by Körber (2018), modified slightly to match the content of the AI banking assistant).*

**Competence**

- I think the AI banking assistant works reliably.
- The AI banking assistant can take on complicated calculations.
- The AI banking assistant could make mistakes. (reverse)
- I am convinced of the skills of the AI banking assistant.

**Understandability**

- The decisions of the AI banking assistant would be understandable and transparent for me.
- The AI banking assistant would do things that I cannot understand. (reverse)

**Intention of Developers**

- The developers of the AI banking assistant are trustworthy.
- The developers of the AI banking assistant take my financial well-being seriously.

**Familiarity**

- I already know similar systems.
- I have used similar systems before.

**Trust in Automation**

- I would trust the AI banking assistant.
- I could rely on the AI banking assistant.

***Intention to use*** *(based on the Technology Acceptance Model TAM3 (Venkatesh & Bala, 2008), modified slightly to match the content of the AI banking assistant).*

- I could imagine using the AI banking assistant in the future.
- I would like to be informed about products that are similar to the AI banking assistant.

***Human-likeness*** *(adapted from Ho and MacDorman (2010), semantic differential scale).*

artificial – natural

synthetic – real

living – inanimate (reverse)

mechanical – biological

***Eeriness*** *(adapted from Ho and MacDorman (2010), semantic differential scale).*

eerie – reassuring (reverse)

predictable – thrilling

bland – uncanny

***Agency*** *(manipulation check).*

- The AI banking assistant has the ability to act in a self-controlled manner.
- The AI banking assistant acts completely independently.

***Personality scales*** *(from the Socio-Economic Panel, Schupp & Gerlitz, 2014).*

**Openness to Experience**

I am someone who …

- is original and brings in new ideas.
- appreciates artistic and aesthetic experiences.
- has a vivid fantasy and imagination.

**Neuroticism**

I am someone who …

- worries often.
- gets nervous easily.
- is relaxed and is able to handle stress well.

**Propensity to Trust**

- One should be more careful with unfamiliar automated systems.
- Automated systems generally work well.
- I trust a technical system rather than distrust it.

**Literature**

C.-C. Ho und K. F. MacDorman, „Revisiting the uncanny valley theory: Developing and

validating an alternative to the Godspeed indices“, *Comput. Hum. Behav.*, Bd. 26, Nr. 6, S. 1508–1518, Nov. 2010, doi: 10.1016/j.chb.2010.05.015.

M. Körber, „Theoretical Considerations and Development of a Questionnaire to Measure Trust

in Automation“, in *Proceedings of the 20th Congress of the International Ergonomics Association (IEA 2018)*, Bd. 823, S. Bagnara, R. Tartaglia, S. Albolino, T. Alexander, und Y. Fujita, Hrsg. Cham: Springer International Publishing, 2019, S. 13–30. doi: 10.1007/978-3-319-96074-6_2.

J. Schupp und J. Y. Gerlitz, „Big five inventory-soep (bfi-s). In Zusammenstellung

sozialwissenschaftlicher Items und Skalen (Vol. 10).“, 2014.

V. Venkatesh und H. Bala, „Technology Acceptance Model 3 and a Research Agenda on

Interventions“, *Decis. Sci.*, Bd. 39, Nr. 2, S. 273–315, Mai 2008, doi: 10.1111/j.1540-5915.2008.00192.x.
